# PEARLS debriefing for social justice and equity: integrating health advocacy in simulation-based education

**DOI:** 10.1186/s41077-024-00320-4

**Published:** 2024-12-18

**Authors:** Niki Soilis, Elizabeth Anne Kinsella, Walter Eppich, Adam Cheng, Lindsay Beavers, Farhan Bhanji

**Affiliations:** 1 Institute of Health Sciences Education, Faculty of Medicine & Health Sciences, McGill University, Montreal, Quebec Canada; 2 Department of Medical Education & Collaborative Practice Centre, Faculty of Medicine, Dentistry and Health Sciences, University of Melbourne, Melbourne, Australia; 3 KidSIM-ASPIRE Research Program, Alberta Children’s Hospital, Calgary, Canada; 4 Departments of Pediatrics and Emergency Medicine, Cumming School of Medicine, University of Calgary, Calgary, Canada; 5 Simulation Program, Unity Health Toronto, Toronto, Canada; 6 Department of Physical Therapy, Temerty Faculty of Medicine, University of Toronto, Toronto, Canada; 7 Montreal Children’s Hospital, Montreal, QC Canada

**Keywords:** Debriefing, Healthcare simulation, Equity, Diversity, Inclusion, Critical reflection

## Abstract

Addressing health inequities in health professions education is essential for preparing healthcare workers to meet the demands of diverse communities. While simulation has become a widely recognized and effective method for providing safe and authentic clinical learning experiences, there has been limited attention towards the power of simulation in preparing health practitioners to work with groups who experience health disparities due to systems of inequality. Balancing technical proficiency with educational approaches that foster critical reflection and inform action oriented towards social accountability is essential. Transformational learning promotes the development of critical consciousness through critical reflection. Debriefing plays a crucial role in fostering learning in this direction by providing a structured opportunity to critically reflect on taken for granted assumptions, examine power and privilege embedded within systems and structures, and empower learners to take action toward changing those conditions. Building on the Promoting Excellence and Reflective Learning in Simulation (PEARLS) Healthcare Debriefing Tool, we propose a PEARLS Debriefing for Social Justice and Equity (DSJE) Tool that specifically directs attention to systems of inequality that contribute to health disparities for vulnerable groups across a range of simulation scenarios. This approach has two aims: (a) to transform debriefings into a critically reflective space by engaging learners in dialogue about social and structural determinants of health that may create or perpetuate inequities and (b) to foster critical reflection on what actions can be taken to improve the health and well-being of identified at risk and vulnerable groups. From this perspective, we can use the adapted PEARLS Tool to incorporate conversations about systems of inequality, equity, diversity, and inclusion (EDI) into our existing educational practices, and make concentrated efforts towards community-driven and socially conscious simulation-based education (SBE).

## From formative to transformative learning in simulation-based education

While simulation-based education (SBE) has become a widely recognized and effective method for providing safe and authentic clinical learning experiences [1–3], there has been limited attention toward the power of simulation in preparing health practitioners to work with groups who experience health disparities due to systems of inequality. Health professions education programs are not only tasked with imparting technical knowledge and skills but also with preparing students to navigate professional practice within a complex societal framework. Balancing technical proficiency with educational approaches that foster critical reflection and inform action oriented towards social accountability is essential. It is important to consider how students can be empowered to manage complex biomedical cases while also developing the ability to identify systems of inequality that contribute to health disparities in historically marginalized and underserved groups.

In the early days of SBE, much of the theoretical foundation in SBE was guided by Kolb’s cycle of experiential learning [4]. Such curricula demand an engineering of appropriately designed learning environments and activities that carefully bridge the gap between learners current knowledge and skills and their next level of capability, all while minimizing unnecessary extraneous cognitive load [5–8]. This focus on mastery performance has led to educational approaches rooted in cognitive psychology, which are effective for teaching procedural skills or for learning to manage emergent situations through deliberate practice [9]. However, when teaching for social justice and equity, approaches that prioritize knowledge acquisition and skill mastery over critical reflection and transformative learning risk reducing these principles to concepts to be learned, rather than values to be understood and enacted [10].

Contemporary educational reforms call for a shift from formative to transformative learning to prepare learners as enlightened change agents who tackle local priorities [11]. The World Health Organization (WHO) has highlighted the importance of preparing future health professionals to deliver service that meets the needs of *both* individuals and communities and that responds to the health inequities experienced by vulnerable groups [12]. Health inequities stem from social disadvantages faced by individuals and groups, in part driven by factors such as systemic racism, socioeconomic challenges, unequal access to education, and limited access to quality healthcare, rather than biological differences [13]. Numerous calls to action have galvanized educational institutions to incorporate the notion of social accountability in the future of health professions education [14, 15]. Nonetheless, incorporating attention to health inequities in SBE has received limited attention, and responding to this call will require a paradigm shift in how we educate healthcare providers of the future.

### Transformative learning through debriefing in simulation-based education: empowering social change

Teaching for social justice and equity requires transformational learning paradigms centered on critical reflection. These paradigms are rooted in critical pedagogies, such as those articulated by Mezirow, Freire, Kincheloe, and Giroux, to name a few [16–19]. Transformational learning promotes the development of critical consciousness through critical reflection. It involves examining taken for granted assumptions, the contextualization of healthcare practices in relation to larger systems, examinations of power and privilege embedded within systems and structures, and the empowerment of learners to take action toward changing those conditions [20–22]. Transformative learning is often stimulated by an experience or catalyzing event where learners face an unfamiliar situation that prompts critical reflection. These events encourage us to examine our own positionality in relation to a situation or injustice, and to uncover submerged power dynamics that benefit some at the expense of others [21, 23, 24].

When thoughtfully designed, SBE can trigger these types of catalyzing events, also known as disorienting dilemmas, which is an experience or realization that challenges previously held assumptions or beliefs about the physical environment, our social interactions, or our feelings and intentions surrounding a difficult experience [16]. This can set the stage for critical reflection, prompting an exploration of assumptions and premises, and an examination of structures and systems that may contribute to health disparities in vulnerable groups. Debriefing plays a critical role in fostering reflection in experiential learning by providing a structured opportunity to reflect on one’s actions, analyze decision-making processes, and identify areas of improvement that can inform future practices [25–27]. Effective communication with peers, mentors, and faculty is a crucial element in this process. In alignment with principles of transformative learning, debriefing is influenced by the communicative process, with interaction, dialogue, and negotiation of shared meaning playing a fundamental role in challenging existing beliefs and the formulation of new perspectives.

Through the creation of a triggering event that prompts critical reflection and communicative learning, SBE can be designed to leverage the transformative potential of an educational experience, making it a suitable pedagogical approach to facilitate dialogue on the social factors that influence patients’ health outcomes. Debriefing can be guided by critical pedagogies and transformative learning theories with the aim of mitigating the hierarchy between teachers and learners and enabling critical dialogue centered on personal and social transformation to help learners become change agents who dismantle harmful systems [28, 29]. These efforts hold promise towards creating critically conscious and socially responsive providers committed to social justice and equity [20, 21].

### Designing the Promoting Excellence and Reflective Learning in Simulation (PEARLS) Debriefing for Social Justice and Equity (DSJE) Tool

Emerging work in healthcare simulation literature contributes to our understanding of both opportunities and potential risks and limitations of addressing equity, diversity, and inclusion (EDI) in SBE [30–38]. This SBE literature is often directed toward specific simulation modalities, such as diversity in the physical simulators we use, or the use of simulated patients to portray underrepresented patient profiles and complex case presentations. While these elements are important and timely, they integrate population-based vulnerabilities and disparities into SBE programming on an intermittent basis, rather than embedding equity conversations within the culture of our simulation practices.

The importance of using SBE to raise awareness of inequities and cultivate interpersonal skills for EDI has been acknowledged [39]. However, concerns about perpetuating stereotypes and biases in case presentations, or disrupting the learning environment, may dissuade some from incorporating EDI-specific curricula until they have more clear guidance on best practices. Despite recognizing the necessity of such curricula, our objective has been to create a universally applicable approach to designing socially conscious SBE programming, regardless of whether the cases are EDI focused or not.

In an effort to use simulation for social justice and equity, the Simulation on a Social Mission (SoSM) initiative was established at McGill’s Steinberg Centre for Simulation and Interactive Learning. This initiative grew out of the observation that vulnerable and underserved populations are underrepresented in health professions curricula. Further, SBE offers unique epistemic conditions, or approaches to constructing knowledge, rooted in the principles of transformative learning that can facilitate critical reflection and dialogue about the socially determined factors that contribute to poor health. The SoSM initiative brought together experts in health professions education, public health, social accountability, and community engagement to develop targeted programs and tools with a focus on vulnerable and underserved patient populations in SBE.

Through consultation with the SoSM interdisciplinary group of experts, we recognized an opportunity to transform the post-simulation debriefing into a critically reflective space for dialogue on: (a) the systems of inequality and (b) the contribution of social and structural determinants to persistent health inequities. Through discussion, we identified the CLEAR (Community Links Evidence to Action Research) Toolkit that provides guidance on initiating conversations about the social determinants of health (SDOH), linking patients to community resources, advocating for change, and facilitating referrals to support services [40]. The CLEAR toolkit was pilot tested among front-line clinicians, who found it to be an effective resource in assessing patient vulnerability, identifying referral resources, and supporting patients in clinical practice [41]. Similar to the PEARLS Healthcare Debriefing Tool, a widely adopted conceptual framework with scripted debriefing in healthcare simulation practices [27], the CLEAR toolkit offered a structured approach to community action by initiating critical reflection and action on SDOH, outcomes we were seeking in the post-simulation debriefing.

Informed by the CLEAR toolkit and the PEARLS Healthcare Debriefing Tool, we created the PEARLS DSJE Tool. This new tool was designed to help facilitators at our Simulation Centre create a supportive environment for critical reflection and dialogue about the social and structural determinants of health and the potential for action oriented toward community health. We sought stakeholder input from education and simulation experts as well as public health experts on the value and applicability of the PEARLS DSJE Tool in SBE. Informal data was collected through consultation with members on the SoSM committee to identify potential issues or challenges that may arise while using the adapted tool during standard debriefings. Stakeholders included two simulation education experts, each with extensive experience deploying SBE, two former program directors in post-graduate medicine, and three professors and curriculum advisors from nursing and physical and occupational therapy, within McGill’s Faculty of Medicine and Health Sciences. These stakeholders represented end users who would integrate this tool into their simulation curricula. We collected and integrated their feedback into refinements of the tool.

Subsequently, the tool underwent informal pilot testing during simulations sessions. We sought input into practical considerations such as faculty usage and its compatibility with the usual debriefing timeframe. A formal pilot test occurred with 4 faculty debriefers and 20 health professions education students through one-to-one debrief interviews following a virtual reality simulation about homelessness. The four faculty debriefers received the tool via email, with scheduled follow-up sessions a few days before the simulation session to address any questions or concerns. According to faculty debriefers, the tool was useful for fostering critical reflection with students, and they appreciated the structured support for the debriefing approach. These additional insights were used to refine the structure and usability of the tool. The student debrief interviews were audio-recorded, with numerous examples of critical reflection on the social and structural determinants of health recorded in the data. This data is currently being analyzed and will be reported separately. We plan broader implementation and the development of a preparatory guide with considerations for the learning environment.

Our modified PEARLS Tool incorporates elements of the CLEAR Toolkit designed to educate and empower health workers to address the SDOH as part of their clinical practice, refer to local support resources, and advocate for wider social change [40, 41]. The PEARLS DSJE Tool integrates the underlying philosophy of referral and advocacy of the CLEAR toolkit, while employing the essence of the original PEARLS Healthcare Debriefing Tool, [27] with the objective, the task, and sample phrases outlined for each step.

We design two specific adaptations to the original PEARLS framework:

- The addition of phase 5: Activism, where educators help name the systems of inequality that lead to the marginalization of groups, leading to increased susceptibility to the presenting problem. This creates the opportunity to facilitate critical reflection on the social and structural determinants of health in relation to the presenting problem by calling attention to how the social context influences management and follow-up needs, or how norms or structures within health institutions can sustain disadvantage. For example, we can consider a patient with diabetes living in a low-income neighborhood in a rural setting. When guiding students to reflect on which patient groups may be more susceptible to the presenting problem, facilitators could initiate dialogue about the prevalence of diabetes among socially disadvantaged groups. Dialogue on the systemic issues of care could address barriers such as inadequate insurance coverage for medications and supplies, transportation issues for clinical follow-ups, and lack of education on self-management skills in a variety of languages and cultural contexts. It could address systemic racism, discrimination and structural inequities, and how these contribute to disparities in diabetes prevalence and outcomes. The debriefing could facilitate critical reflection on how socially rooted circumstances contribute to health outcomes, including barriers to preventive care and healthy lifestyle choices; socioeconomic factors that perpetuate inequities; and the interplay of discrimination, racism, and unequal access to health services. This phase is designed to enhance the learner’s awareness and readiness to care for complex and underserved patient groups by educating learners to develop a holistic care plan that takes into account the patient’s physical and psychosocial circumstances.
- The update of phase 6: Application Summary with a community-focused theme that ties learners to the larger health system. The goal is to explore the availability and limitations of referral and support networks in the community for vulnerable and underserved groups and to understand how systems of inequality impact those supports. It provides prompts on potential actions to improve the health and well-being of identified at-risk and vulnerable groups. Referring back to the example of a patient with diabetes living in a low-income neighborhood in a rural setting, various resources can be explored to support the patient’s health management and address the biopsychosocial factors impacting their condition. These resources could include virtual visits, peer support groups, nurse practitioner follow-ups, referral to interdisciplinary services and community health programs promoting nutrition, social support, and healthy lifestyles. In addition, raising critical consciousness about issues of equity in the formative stages of professional development, position future healthcare practitioners to become transformative agents of social change in underserved communities and to become advocates for systemic changes toward proper infrastructure for community green spaces, sustainable food sources, social housing, mobile health clinics, clean water and sanitation, and much more.

**Fig. 1:**
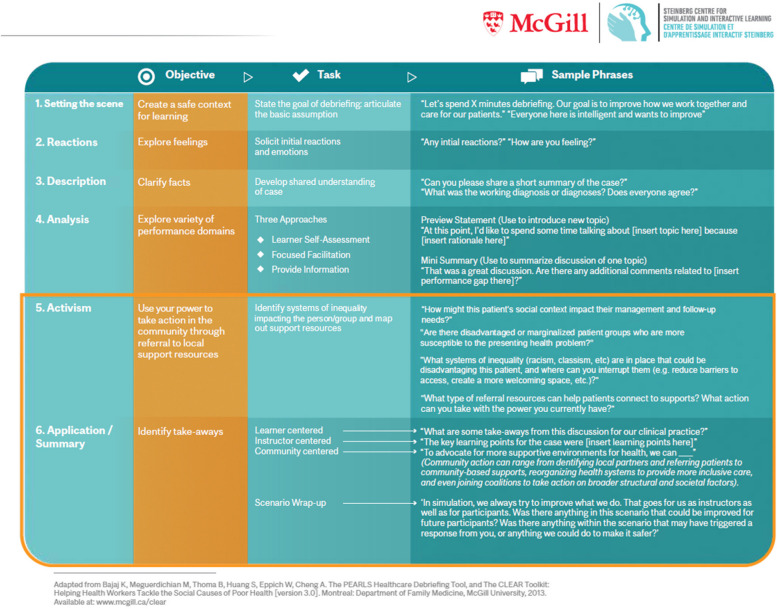
PEARLS DSJE Tool

These additions create a systematic approach to leveraging the power of debriefing through a health equity lens. Further details on the questions and prompt are outlined in Fig. 1 PEARLS DSJE Tool below.

### Future of simulation for the realities of the twenty-first century

Simulation and debriefing are powerful strategies to promote critical reflection on the social and structural determinants of health influencing health outcomes. However, the use of SBE to facilitate dialogue around equity and social justice remains limited. Although critical pedagogies in simulation have been explored [42–46], these approaches have yet to be integrated into SBE in a concerted and scholarly way. The PEARLS structured debriefing framework has been expanded to debrief simulations pertaining to interprofessional team dynamics and system-focused threats [47–50]. However, we see a clear need to position the PEARLS approach as a means to engage learners in dialogue about social justice and equity issues in the communities they serve. The PEARLS DSJE Tool fills this gap by building on the original PEARLS debriefing tool. Our modified PEARLS Tool can be used across a range of scenarios to specifically facilitate transformational dialogue that names systems of inequality in relation to the clinical problem and their patients. This approach may raise awareness of power, privilege, and oppression in our communities and engage learners in critical reflection on the social structures and institutions that create or sustain disadvantage. Importantly, our tool can help learners identify concrete actions they can take to improve the care of their communities. Future research should engage SBE experts to gather additional input and feedback on the use of this tool and contribute to best practices on its effective and impactful implementation in different simulated environments. Research focusing on the attitudes, behaviors, and impact of employing this tool to promote social justice, equity, and activism following SBE sessions would be invaluable. Additionally, exploring learners’ and faculty perceptions of the tool’s utility and effectiveness, as well as perceptions regarding its role in facilitating critical reflection on social determinants influencing health outcomes, is essential.

## Data Availability

Not applicable.

## Abbreviations

CLEAR Toolkit: Community Links Evidence to Action Research Toolkit
CLT: Cognitive load theory
EDI: Equity, diversity, and inclusion
PEARLS Tool: Promoting Excellence and Reflective Learning in Simulation Debriefing Tool
PEARLS DSJE Tool: PEARLS Debriefing for Social Justice and Equity Tool
SBE: Simulation-based education
SoSM: Simulation on a Social Mission
